# Assessing human-bat interactions around a protected area in northeastern Brazil

**DOI:** 10.1186/s13002-015-0058-7

**Published:** 2015-11-17

**Authors:** Karlla Morganna da Costa Rego, Caio Graco Zeppelini, Rômulo Romeu Nóbrega Alves

**Affiliations:** Programa Regional de Pós-Graduação em Desenvolvimento e Meio-Ambiente – PRODEMA, Universidade Federal da Paraíba, Campus I, Cidade Universitária, João Pessoa, 58051-900 PB Brazil; Programa de Pós-Graduação em Ciências Biológicas – Zoologia, PPGCB-ZOOLOGIA, Universidade Federal da Paraíba, Campus I, CIdade Universitária, 58051-900 João Pessoa, PB Brazil; Departmento de Biologia, Universidade Estadual da Paraíba, Avenida das Baraúnas, 351, Bodocongó, Campina Grande, Paraíba CEP 58109-753 Brazil

**Keywords:** Bats, Ethnozoology, Folklore, Human-nature interactions, Traditional medicine, Morcegos, Etnozoologia, Folclore, Interações humanos-natureza, Medicina tradicional

## Abstract

**Background:**

Bats are key components to the Neotropical forests. Unfortunately, their bad reputation is a major obstacle in their conservation as it creates fear and hostility towards them. Understanding this reputation acquired by bats and studying interactions between bats and humans has shown fundamental promise when creating strategies to forge a non-antagonistic coexistence between both parts and in the promotion of bat conservation in areas with ever-rising human occupation.

**Methods:**

Ninety people were surveyed from three villages that were situated around a Biological Reserve in the state of Paraiba; located in Northern Brazil. The survey was completed using semi-structured interviews addressing villager’s knowledge of the biology and ecology of bats, their interactions with bats, potential medicinal uses, and their socioeconomic situation. Additionally, we sampled the bats that reside in or visit these villages.

**Results:**

Bats were often considered harmful, dangerous and carriers of disease. Bats were often connected to hematophagia, as well. The respondents believe that impacts such as the deforestation are forcing bats into urban environments. With this research, we were able to register one of the few records of bats in popular medicine in Brazil.

**Conclusion:**

The folklore and superstition surrounding bats can form an obstacle that affects their conservation. Environmental education is an important step in order to create a harmonious coexistence between humans and bats and to mitigate the impending conflicts between humanity and nature.

## Resumo

**Introdução:**

Morcegos são componentes-chave da fauna das Florestas Neotropicais, mas carregam uma imagem ruim que gera medo e hostilidade contra eles e que se faz um empecilho para sua conservação. Entender a visão e conhecimento das populações tradicionais sobre os morcegos é fundamental para criar estratégias visando coexistência pacífica e promover a conservação dos morcegos em áreas com crescente ocupação humana.

**Métodos:**

Entrevistamos 90 pessoas de três vilarejos no entorno de uma Reserva Biológica no norte do estado da Paraíba usando de entrevistas semiestruturadas questionando sobre o conhecimento dos entrevistados na biologia e ecologia dos morcegos, as interações entre ambos, possíveis usos místicos ou medicinais, assim como a situação socioeconômica dos participantes. Foi realizada junto uma amostragem das espécies de morcegos que ocorrem nos vilarejos.

**Resultados:**

Os morcegos foram comumente citados como perigosos ou danosos, associados a doenças, e o estigma de “sugadores de sangue” é generalizado. Os entrevistados consideram que os impactos ambientais, como o desmatamento, são responsáveis por levar os morcegos para dentro dos ambientes urbanos. Nós também registramos um dos primeiros relatos de uso de morcegos em medicina popular no Brasil.

**Conclusão:**

Os morcegos são animais ainda imersos em folclore e superstição, o que pode ser um obstáculo para a conservação. Educação ambiental é um passo importante para criar uma coexistência harmoniosa entre humanos e morcegos, e mitigar possíveis conflitos humano-natureza.

## Background

Bats are mammals of the order Chiroptera; the second most diverse order among mammals. They play remarkable roles in tropical ecosystems. In addition to seed dispersal, pollination, forest regeneration and balanced control of arthropod populations [1, 2], bats emerge as high-quality bioindicators, as a response to an array of complex human habitat alterations and developed responses linked to other taxa, as well [3].

Although their role in the ecosystem is key, most people have little opportunity to observe and understand the essential behaviour and biology of bats. This lack of understanding is attributed to several causes. In Brazil, two foremost reasons are that the majority of the population lives in urban centres that are distant from most wildlife [4] and a lack of affective attitude or appeal toward bats [5]. The array of myths and folklore in Western Culture, together with the lack of information about bats, tends to taint their image, erasing their role on environmental health and becoming an obstacle in the way of responsible behaviour toward these animals [6, 7]. As a result, bats end up being treated as pests or dangerous animals that must be exterminated [8, 9]. Although many cultures in East Asia perceive bats as good omens [10], they are mainly portrayed as conveyors of death, disease and dark forces, in the myths and tales of Western civilizations [11]. In this context, bats and humans are presented with a conflict [12] that is an issue of grave concern regarding conservation activity. Although rarely taken into account when these conflicts are analysed, social factors can play a prominent role in such conservation concerns [13].

When it comes to the collision of humans and nature, it is fundamental to incorporate human perceptions and their interactions with nature into research by considering the societal aspects and any damaging cultural consistencies among the human inhabitants of the region, with an ethnozoological approach [14–16]. Using ethnobiological studies, researchers can create a basis for developing specific actions towards human-wildlife conflicts, gather information about the necessities, activities, and specific impacts of a community [17, 18] in an instrumental effort in mitigating the potential conflicts; especially within communities that are in closer contact with wildlife and have limited access to information [19], for they have a greater chance to enter in contact with bats, but lack ways to mitigate any risks associated with this contact.

This paper discusses an initial assessment of the relationship between humans and bats. In a field survey, the inhabitants of three villages of a Biological Reserve in Paraíba, Brazil, were asked about their perception of bats and their understanding of the roles that bats play in nature.

## Methods

**Fig. 1:**
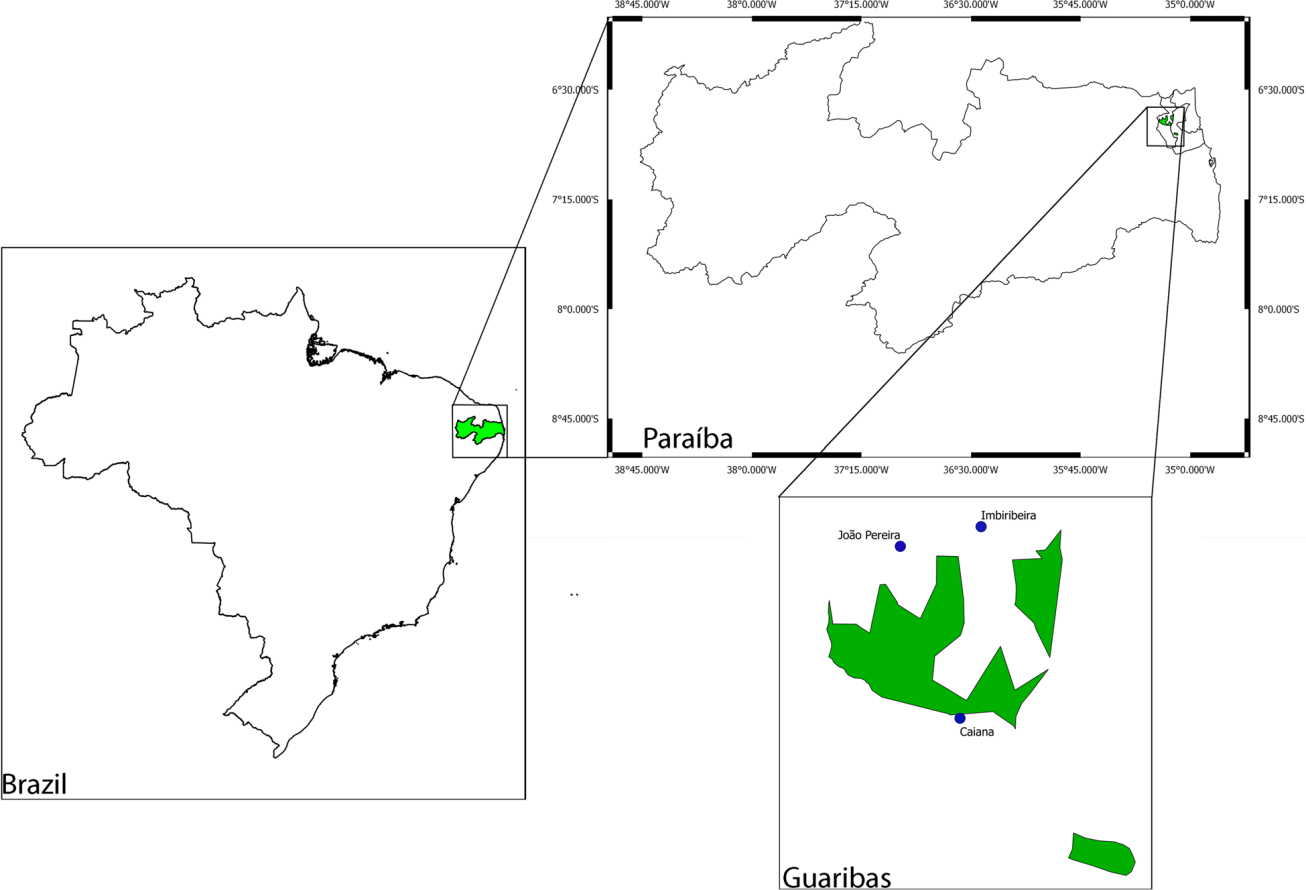
Location of the villages visited during the study. The villages are located in the municipalities of Mamanguape and Rio Tinto, northern Paraíba state, Brazil. JP stands for “João Pereira”, IMB for “Imbiribeira”, and GUARIBAS is the Biological Reserve, with its area represented in the smaller scale, in green

Inhabitants of three villages, Caiana, Imbiribeira and João Pereira were interviewed. These villages are situated around the perimeter of the Guaribas Biological Reserve; a federal conservation unit located in the municipalities of Mamanguape and Rio Tinto in the state of Paraíba (Fig. 1). The villages are in close contact with the reserve. Some residents of these villages live as close as 5 meters from the reserve’s fences. The villages are small sub-urban agglomerations, lacking a considerable amount of modern infrastructure; such as treated water supplies and paved streets. This population relies heavily on subsistence crops and livestock (cattle, goats, chickens). It is important to note that there is no official data on the population and demographics of the villages studied.

A questionnaire was developed as a tool to standardize semi-structured interviews [20, 21]. The main goal of the questionnaire was to comprehend the relationship between the residents and their local bat fauna. The questionnaire addressed their knowledge about bat biology (diet, habitat, behaviour), their roles in nature and public health, and perceived medicinal or mystical uses. Additionally, information about the socioeconomic status of each participant was collected. The questions were either discursive or multiple choice, according to the information expected.

The interviews were initially deployed using a tape recorder to catalogue the conversation between the researcher and participant. Due to the intimidation or inhibition that some respondents experienced with the presence of a recorder, we adopted a direct transcription method; annotating the answers that the subjects provided on paper. All interviews were held under a Term of Ethics (Termo Livre Esclarecido) granted by the Ethics Committee of Universidade Federal da Paraíba (UFPB). The inquired subjects were free to answer the questions in their own ways; sometimes generating more than one answer to the same question, due to that some of the results sum up more answers than interviewed people. To complement the study of the human residents of this area, we performed an inventory of the diversity of bats that inhabit and/or visit the villages. Samples were collected for three nights of each month over a period of 12 straight months. Following the methodology recommended by Flaquer et al. [22], ground level mistnets were installed within the village’s limits and were opened at dusk then closed at midnight. The bats that were captured were taken to a laboratory for taxonomical identification and deposited in the Coleção de Mamíferos of Universidade Federal da Paraíba. Following the model of multiple competences [23, 24] in which all information supplied is taken into account, the data were analysed and presented through graphics generated by KyPlot [25].

We interviewed 33 inhabitants of Imbiribeira, 29 of Caiana and 28 of João Pereira, summing up 90 people (45 male, 45 female). The demographics of the respondents are summarized in Table 1 (note that 1 Brazilian Minimum Wage = 764 BRL). We captured 650 specimens, representing 14 species of 12 genera and three families (Table 2).

**Table 1:** Demographic summary of the respondents

		Female	Male
Age	18–25	12	13
	26–33	10	14
	34–41	7	6
	41+	16	12
Income (Minimum Wages)	1	19	18
	2	19	17
	3+	7	10
Education	Illiterate	3	3
	Incomplete elementary	20	13
	Elementary	9	9
	Incomplete High School	11	10
	High School	2	10

**Table 2:** Bat species sampled using mist-nets at the villages

Family	Species	Common name	Abundance
Emballorunidae	*Sarcopterix leptura*	Lesser Sac-winged Bat	1
	*Peropteryx leucoptera*	White-winged Dog-like Bat	1
Mormoopidae	*Pteronotus personatus*	Wagner’s Mustached Bat	1
Phyllostomidae	*Carollia perispicillata*	Seba’s Short-tailed Bat	110
	*Desmodus rotundus*	Common Vampire Bat	21
	*Glossophaga soricina*	Pallas’s Long-tongued Bat	1
	*Lophostoma brasiliense*	Pygmy Round-eared Bat	1
	*Phyllostomus discolour*	Pale Spear-nosed Bat	2
	*Artibeus planirrostris*	Flat-faced Fruit-eating Bat	365
	*Artibeus lituratus*	Great Fruit-eating Bat	50
	*Artibeus obscurus*	Dark Fruit-eating Bat	13
	*Dermanura cinerea*	Gervais’s Fruit-eating Bat	47
	*Plathirrynus lineatus*	White-lined Broad-nosed Bat	17
	*Sturnira lilium*	Little Yellow-shouldered Bat	20

## Results

When questioned about the bat diversity, 50 (55 %) of the respondents declared to know or recognize the existence of various bat species or varieties, but had no means to differentiate or describe the variety. A total of 22 (25 %) respondents pointed out that they recognized two species, the “Rampa” and the “mirim”, 3 (4 %) said that there were the “black bat” and the “striped bat”, the remaining 14 (16 %) had no answer to the question.

**Fig. 2:**
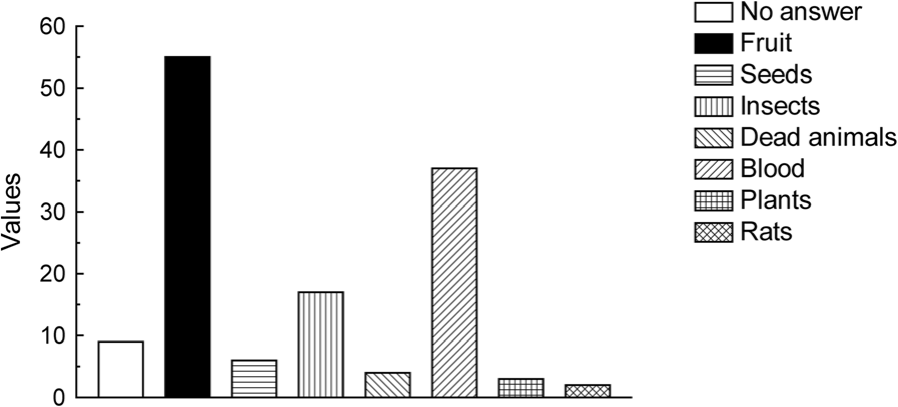
Food items consumed by bats, as stated by the respondents, expressed in absolute values

When questioned about their perception of bats as “good” or “bad” animals, 82 (91 %) respondents reported bats as bad or negative, only 8 (9 %) said that they were good. In the interviews, there was a general belief that bats are evil or malicious entities. Regarding diet, 55 (61 %) respondents answered that bats are frugivorous, 37 (41 %) said blood, and various other food items were also cited (Fig. 2). About their roosting habits, the most common answers were human structures (i.e. roofs, bridges, abandoned buildings) with 68 answers, then trees as the second most common response (46). Seventeen people broadly answered, “the woods”, and five had no answer. On their activity period (“when do bats leave their roosts?”) 80 respondents (89 %) answered that they start activity by nightfall.

On the difference between the number of bats seen in the villages today compared to years ago, 28 (31 %) respondents answered no noticeable change on their abundance, 27 (30 %) said that they have become more visible, and 16 (17 %) pointed to a decrease on their abundance. Upon further questioning about why the bats were increasing abundance in the villages, the respondents mentioned deforestation as a factor; pushing them into the urbanized areas. Another response was that their crops are a source of resources that attract bats into the villages. Those who said that the bat abundance was declining also pointed the deforestation (loss of habitat and roosts) and the pesticides used in the crops.

**Fig. 3:**
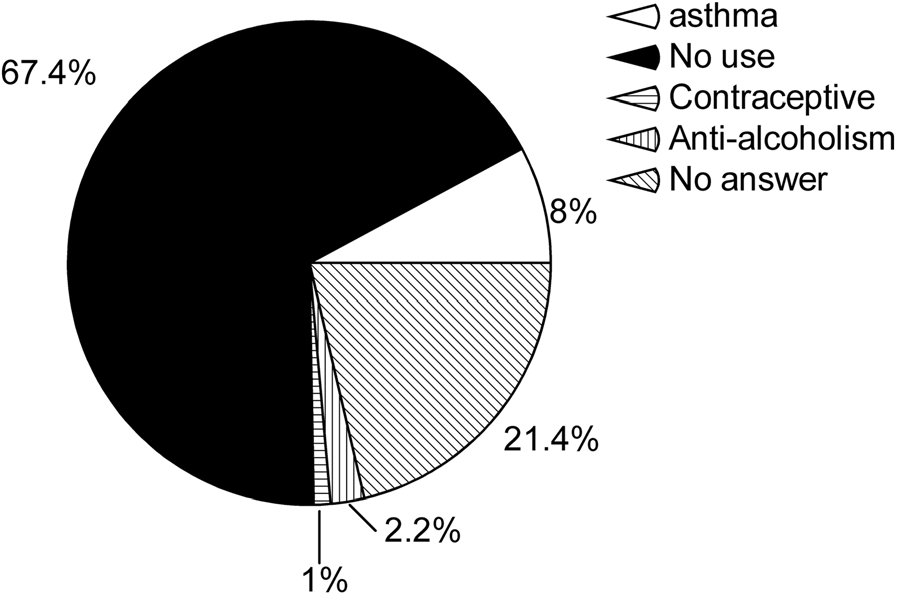
Uses of bats in popular medicine, expressed in percentage of the answers

**Fig. 4:**
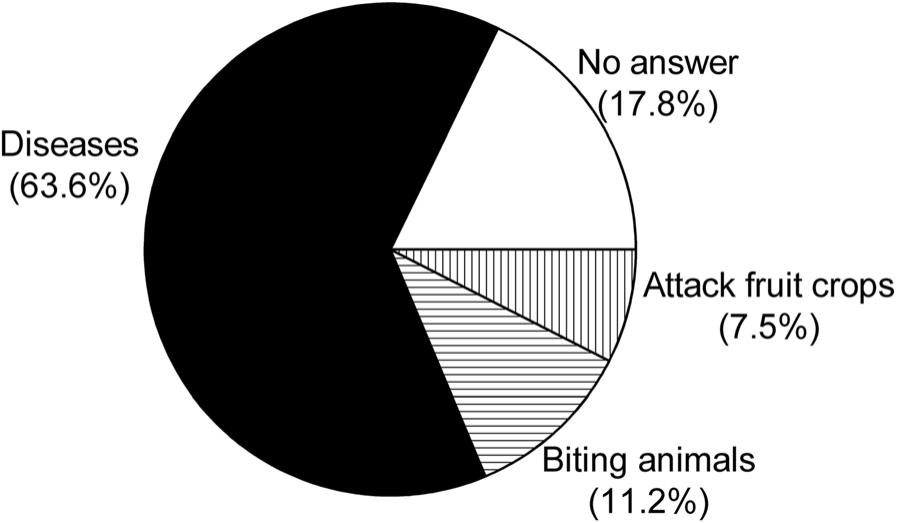
Potential harm bats can cause to humans, expressed in percentage of the answers

About the use of bats in traditional medicine, 60 (67.4 %) of the respondents said they were of no use, and 19 (21.4 %) had no answer. Yet, 10 (11.2 %) mentioned uses on the treatment for alcoholism, asthma and as a contraceptive for livestock (Fig. 3). For the medicinal uses, the bats are roasted and then pulverized into ashes. On the potential harm or personal loss caused by bats, 68 (63.6 %) of the respondents pointed out transmission of diseases through the faeces or urine, rabies included; other answers involved biting household animals and livestock to suck blood and attacking the fruit crops (Fig. 4). Yet, it is important to mention that we registered answers that added information about potentially good roles of bats, as predators of unwanted insects and seed dispersers, as well.

## Discussion

The taxonomic approach is the first step needed to process and create information from data obtained from traditional knowledge; as traditional communities normally drive their attention towards what is interesting or useful to them, hence it can be expected that fauna with some instrumental value will be better understood [26, 27]. The bat sampling that was performed in the villages registered 14 species. Nevertheless, the majority of the respondents expressed that they knew more than one species but they were not able to distinguish between more than two species or “kinds”; a fact that is understandable, as bats are not of interest or utility to their daily life. It has been confirmed that wide array of species might be referred to using a single term (such as “bats”, in our case) by traditional communities when they are not of interest or use to the villagers; in contrast, these communities may notice greater differentiation in more popular taxa [28]. An example can be seen on the Peruvian Amazon Matzes, who use a single name for over 100 species of bats [29].

The respondents reported four ‘varieties’ of bats: the “rampa”, the “mirim”, the striped bat and the black bat. Both the “rampa” and the striped bat might refer to “Great Fruit-eating bat” *Artibeus lituratus*, matching the description of a large bat with facial stripes and being very common in the area. We believe it is unlikely that they refer to a closely related species, the “Flat-faced Fruit-eating bat” *Artibeus planirostris* (also abundant in the area) because the facial stripes of the species are commonly faded, matching the colour of the rest of the fur. The “mirim” and the black bat seem to represent a small sized insectivorous bat. It is likely that these bats members of the families Vespertilionidae, Molossidae, Emballonuridae or a small member of Phyllostominae (such as *Micronycteris*); each family matching the description of a small, fast flying bat that feeds on flying insects.

Among the Chiroptera order, that amounts to over 1120 known species [30], there are only three species of hematophagous bats (“Common Vampire bat” *Desmodus rotundus,* “Hairy-legged Vampire Bat” *Diphylla ecaudata* and “White-winged Vampire Bat” *Diaemus youngi*). Despite this fact, nearly half of the respondents in our study area referred to bats as blood feeders. Additionally, many of them associated this dietary habit with the transmission of diseases, especially the rabies virus. The bat fauna samplings performed in this study registered only 21 individuals of *D. rotundus* from 650 individuals captured. The fact that over 90 % of the respondents characterized bats as bad animals, despite the small number of blood-feeding bats in their area, illustrates how deeply the reputation of bats as vicious creatures that attack humans to suck blood is rooted. It is important that the villagers understand that the transmission of rabies is not limited to bats. Any infected mammal can transmit the rabies virus through bite or contact with bodily fluids [31, 32]; excluding faeces or urine, as suggested by some of the respondents. Some of the survey answers revealed a misguided notion that bats have a habit of eating dead animals and carcasses and were, subsequently, linked to being highly infected with microbes. Although bats might have the widest trophic versatility among the vertebrates, feeding on nectar, fruits and flowers, insects, blood, fish and small vertebrates [33], there is no record of bats feasting on carcasses.

Bats are known to be able to utilize human structures as roosts, with some having the capacity to adapt to human-altered landscapes where building and other structures are placed (e.g. roofs, concrete pipes, and bridges). This allows bats to acclimate to urban landscapes [34, 35]; hence being able to survive in human occupied areas as they lose their habitat. This attribute allows bats to be present and abundant in the villages that were studied. When questioned about the presence and abundance of bats in the villages the respondents mentioned factors that are related to habitat loss and human alteration on their landscape as causes of bat invasion of anthropic areas. In contrast, this was described as a cause for their decline in abundance, as well. Deforestation is a major cause of biodiversity loss [36], with a good portion of the deforested area being substituted by urban landscape [37], in which a part of the original pool of species might still be able to survive, bats included [38]. Among the bats that were sampled in the villages the majority were frugivorous species, like *Artibeus planirostis, A. lituratus* and “Seba's Short-tailed Bat” *Carollia perspicilatta*. These species are capable of using crops as a food source, and are also known to obtain food from ornamental trees and gardens in human occupied areas [39, 40]. Although registered in low abundances, four species of insectivorous bats (“Lesser Sac-winged Bat” *Saccopteryx leptura,* “White-winged Dog-like Bat” *Peropteryx leucoptera,* “Wagner's Mustached Bat” *Pteronotus personatus* and “Pygmy Round-eared Bat” *Lophostoma brasiliense*) and one omnivorous species that include insects on their diets (“Pale Spear-nosed bat” *Phyllostomus discolour*) are species that are often attracted to human occupations in order to forage around the artificial lights where insects can be found [41]. Bats are commonly undesired guests in human altered landscapes due to a combination of their bad reputation, the perceived threat to personal gardens and orchards, and their use of roofs as roosts. Roosting on roofs is undesirable for villagers because it is common for bat faeces and urine accumulate and cause strong odours, along with the possibility of disease transmission [42].

Bats commonly feature in folklore, with many legends about them. The most recurring are that bats are old rats that develop wings and that all bats are vampires [19]. In our study, the respondents had not cited this myth, but they had referred to others, such as bats having ‘insect blood’ and that their bite can cause lethargy to the extent that it will make the bitten individual stop eating. Additional myths included bats being unable to see in daylight, bats foraging on carcasses and decomposing animals and bats having an evil eye. Myths can inspire a bad reputation for bats, causing aversion or fear towards them and potentially posing a threat to them, inspiring extermination campaigns and making people oppose or refuse to cooperate in conservation or protection programs. A conscientious society must promote conservation without prejudice, protecting not just the charismatic or directly useful fauna, but aiming to protect the ecological balance [43].

There is a vast knowledge of the uses of Brazilian fauna and flora in popular and traditional medicine [44–46]. Moreover, there are reports of the consumption of bats as medicine and bushmeat worldwide [47–49]. Our work is one of the few reports of bats being used in popular medicine in Brazil. The only other published registry was by Ferreira et al. [50] where *Desmodus rotundus*’ medical uses were identified by street-fair merchants in Salvador/BA. The consumption of wildlife as medicine or a food source must be considered a concern of public health, as most emerging infectious diseases are zoonotic [51] and because humans might be consuming or manipulating species that are natural reservoirs [52–54]. This is dangerous because it brings the human populations in contact with the transmission cycle of wild pathogens. Bats are back in the spotlight with the recent Ebola outbreak in West Africa. Bats are known to be efficient reservoirs for the virus and it is known that the consumption of infected bats can be a transmission pathway for the illness [55–58].

In Brazil the law 5197/1967 rules that all autochthone fauna and their ecological and spatial niches are under government protection; their persecution, hunting or destruction are forbidden and punishable [43]. Yet, bats have traditionally been regarded as dangerous and exterminated in many areas [8]. At the end of each interview, we handed out a pamphlet containing information about bats, their importance to the environment and additional information on how to proceed in any case of contact. This was done in an effort to transfer unbiased information about bats and to attempt to break their bad image. The pamphlet was also the instrument that we created to narrow the gap left by the absence of accessible informative material within the community. The pamphlets were an opportunity to engage with the respondent in a conversation where additional information about bats could be offered in an informal way, creating a more relaxed atmosphere when raising awareness to the subject.

## Conclusion

Bats are an animal group steeped in folklore, with many legends about them. Given their bad reputation, bats might suffer extermination campaigns and people might oppose or refuse to cooperate in conservation or protection programs. We campaign for the necessity of environmental education as an important step in order to break the negative misconception and prejudice that bats face. Such actions can only succeed if a sound knowledge basis of human-nature relations exists.
