# Erratum to: Assessing human-bat interactions around a protected area in northeastern Brazil

**DOI:** 10.1186/s13002-015-0069-4

**Published:** 2015-12-29

**Authors:** Karlla Morganna Costa Rego, Caio Graco Zeppelini, Luiz Carlos Serramo Lopez, Rômuno Romeu Nobrega Alves

**Affiliations:** Programa Regional de Pós-Graduação em Desenvolvimento e Meio-Ambiente – PRODEMA, Universidade Federal da Paraíba, Campus I, Cidade Universitária, João Pessoa, 58051-900 PB Brazil; Programa de Pós-Graduação em Ciências Biológicas – Zoologia, PPGCB-ZOOLOGIA, Universidade Federal da Paraíba, Campus I, CIdade Universitária, 58051-900 João Pessoa, PB Brazil; Departmento de Biologia, Universidade Estadual da Paraíba, Avenida das Baraúnas, 351, Bodocongó, Campina Grande, Paraíba CEP 58109-753 Brazil

## Erratum

An author was unfortunately omitted from the original publication of this article [1]. Luiz Carlos Serramo Lopez has been added as the third author of this article and the Authors’ contributions section has been updated accordingly.
